# Will Green CSR Enhance Innovation? A Perspective of Public Visibility and Firm Transparency

**DOI:** 10.3390/ijerph15020268

**Published:** 2018-02-04

**Authors:** Weiwei Wu, Yexin Liu, Tachia Chin, Wenzhong Zhu

**Affiliations:** 1 School of Management, Harbin Institute of Technology, 13 Fayuan Street, Nangang District, Harbin 150001, China; wuweiwei@hit.edu.cn (W.W.); liuyexin1990@163.com (Y.L.); 2 School of Management, Hangzhou Dianzi University, Xiasha District, Hangzhou, 310018, China; 3 School of Business, Guangdong University of Foreign Studies, 2 Baiyun N Ave, Guangzhou District, Guangzhou 510420, China; wenzhong8988@sina.com

**Keywords:** green CSR, public visibility, firm transparency, innovation performance, moderating effect

## Abstract

In response to the asking and requiring of stakeholders to be more environmentally responsible, firms must commit to green corporate social responsibility (CSR). Firms being green and responsible always can acquire intangible resources that are important for firm innovation. Given the scarcity of existing research addressing relevant issues in depth, this paper expands our understanding of green CSR by revealing its antecedent effects on firm innovation performance. We also include public visibility and firm transparency as contingency factors to explore the relationship between green CSR and firm innovation performance. Using data collected from publicly listed firms in China, we find that greater innovation performance is associated with an increase in firm green CSR, and the positive relationship between green CSR and innovation performance is moderated by public visibility and firm transparency. Based on the results, theoretical contributions and practical implications are outlined.

## 1. Introduction

With the rise of environmental awareness, global organizations have increasingly been required to attach importance to environmental problems [1,2]. To face the increasing emphasis on environmental protection from stakeholders, firms make active efforts to involve environmental concern into their corporate landscapes and strategic decision-making processes, in an attempt to go green [3]. As such, more and more firms have started to involve environmental protection actions into the implementation of corporate social responsibility (CSR) [4], which results in the emergence of the concept “green CSR”.

Green CSR is actually an essential and different aspect of CSR [5]. CSR is a firm’s multidimensional voluntary activities toward different stakeholders [6], which is beyond the interests of the firm [7]. Green CSR reflects firms’ initiatives that are good for the environment rather than simply complying with the related laws and regulations [8]. Despite linking environmental management to CSR, research by Baughn et al. [9] indicates that compared to firms in other countries, US businesses score higher than expected in CSR but score lower than expected in green CSR. In other words, higher levels of CSR do not always produce better green CSR. While environmental performance on reducing waste and emissions has become strategically essential for business production and operation, green CSR has drawn considerable attention of practitioners and evolved to be a critical topic in academics nowadays.

More and more literature studies the reasons why firms are committed to green CSR and how it is conductive to firm performance, as summarized in Table 1. Scholars mostly argue that performing green CSR can upgrade competitiveness, enabling firms to accumulate a competitive advantage [10,11] by reducing environmental costs (e.g., waste reduction, energy/water use, penalty expenditure), increasing marketing revenues (e.g., increasing customer recognition, company reputation and/or brand loyalty), or bringing financial profit (e.g., ROA, ROI).

Despite numerous researches approach the relationships between green CSR and performance using different performance measures (e.g., environmental, marketing, and financial), there is still a dearth of research discussing the mechanisms between green CSR and innovation performance. From the theoretical perspective, being green is a catalyst for innovation; coping with environmental regulations requires the development and/or the adoption of new technologies to create favorable conditions for firms to trigger potential innovation [19,20,21]. Following this logic, there is supposed to be a close link between green CSR and innovation performance. However, the question of whether green CSR translates into innovation performance remains unclear. Without a proper understanding of this important question, firms are unable to derive appropriate strategies for enhancing green CSR. As such, this paper endeavors to probe into the new field of study, focusing on demonstrating the associations between green CSR and innovation performance.

Furthermore, as proposed by the stakeholder theory, firms are not self-contained or self-sufficient but are dependent upon their various stakeholders for ensuring a continuous inflow of resources and support [22]. The increased level of environmental awareness creates the need for firms to adapt to stakeholders’ demands. When stakeholders do not have as much information about firms’ green CSR practices, information asymmetry occurs. When stakeholders communicate with firms frequently, they trust the quality of and construct a shared vision about the firms’ green CSR disclosure. By developing a shared vision, firms can give meaning to their stakeholders and energise them to commit to resources and support supply. Hence, the correlation between green CSR and firm performance might depend on the existence of a shared vision between firms and stakeholders. Public visibility and firm transparency are two important ways for firms to create a shared vision with stakeholders. Therefore, this paper further investigates the correlation between green CSR and innovation performance using public visibility and firm transparency as contingency factors.

This paper brings implications for the unexplored mechanisms between green CSR and innovation performance. Specifically, we address two research questions: (1) Is green CSR associated with greater innovation performance? and (2) Does the relationship between green CSR and innovation performance differ for firms with higher public visibility and firm transparency? These research questions are tested in the context of a transition economy—China. Although the conceptual arguments explored in this study are quite general, a transition economy provides a useful sociopolitical context. Chinese firms are highly dependent on the government and China’s market seem quite diverse, which allow us to observe large variations in some of the specific factors potentially relevant to the relationship between green CSR and innovation performance.

The remainder of the paper is structured as follows. The following section provides arguments about the relationship between green CSR and innovation performance, and delineates the moderation effects of public visibility and firm transparency on the correlation between green CSR and innovation performance. Section 3 addresses the method issues, including data collection and variables measurement. Section 4 reports the analysis results and discusses them. Section 5 concludes main findings, discusses theoretical contributions and practical enlightenment, and provides future research agendas.

## 2. Hypotheses Development

### 2.1. Green CSR and Innovation Performance

Green CSR is the recognition of obligation or the waste-reduction practice of firms’ operation to maximize the efficiency of their inputs and minimize the means of negatively influencing the future generations of the country [23]. In recent years, worldwide environmental issues have brought an increasing challenge to firms that used to pursue fast growth at the expense of massive resource consumption and environmental degradation. Therefore, green CSR is gaining more importance due to the changes of stakeholders’ values from profit-oriented to ecology-friendly. In order to cater to global stakeholders’ interests, today’s firms are often compelled to behave more responsibly toward the environment. Previous research [24,25] points out three critical stakeholder groups that may act as a driving force to propel firms to undertake green CSR, which are community stakeholders such as industrial associations and non-profit organizations, regulatory stakeholders such as governments and legislatures, and organizational stakeholders including employees, partners, and media. The first two groups are among the most important stakeholders giving attention to firms’ implementation of green CSR [26].

Following the foregoing arguments, it can be inferred that firms with more emphasis on green CSR issues may gain more positive feedback from all kinds of stakeholders, particularly those community stakeholders such as non-profit green groups, as well as regulatory ones. Firms focusing on environmentally friendly activities can capture valuable information and knowledge about the community stakeholders’ green needs, preferences, and early warnings about shifts in green values [27]. The external information and knowledge accepted by collective communication with community stakeholders can help firms achieve innovative potential, as firms can embody this information and knowledge in innovation processes [28]. In this sense, firms with stronger green CSR can lead to innovation through improving the quality features of their products with environmental concern, which may enable their offerings to be unique [29]. Thus, green CSR initiative may be the origin station of proactive innovation. For instance, Jacobs et al. (2010) find that announcements of philanthropic gifts for environmental causes are associated with significant positive market reaction [30]. Wei et al. (2017) argue that green CSR signals a firm’s efforts to accommodate pressure from business stakeholders, and thus green CSR indirectly influences firm performance through business legitimacy [18]. These empirical results support the argument that firms implementing green CSR could establish cooperation relationship with community stakeholders, which further leads to knowledge input and trigger innovation.

More importantly, by covering the negative impacts of products, operations, and facilities on the environment, firms undertaking green CSR also obtain potential benefits form regulatory stakeholders’ supports, including financial supports and political supports to promote their innovation activities. As the most influential stakeholder, regulatory stakeholders have the power to channel valuable resources toward or away from a firm [31]. Regulatory stakeholders have constructed general environmental protection codes and norms together with related incentive schemes. Firms demonstrate that their obedience to these codes and norms may contribute to increase their political legitimacy from the perspective of regulatory stakeholders, thus resulting in active responses [32]. Therefore, firms that limit their adverse environmental impact can obtain financial capital and preferential political support form regulatory stakeholders, such as tax exemption, support funding, project subsidy, interest-free or discount government lending, and relaxed or stiffer regulatory enforcement. These financial capital and preferential political supports are critical for firms seeking to build up their R&D base, implement their R&D projects, and upgrade the skill level of their R&D employees with the potential to generate high innovation performance. Previous research also stresses the impact of green CSR on political legitimacy, which can be taken as a signal of support for our arguments. For instance, Babiak and Trendafilova (2011) indicate that environmental management initiatives in business are important to acquire legitimacy [33]. Yang et al. (2015) reveal that legitimacy plays a mediating role in the link between adoption of green management practices and competitiveness [34].

Some aspects of the stakeholder view are actually in concert with the resource-based view (RBV), as green CSR can be seen as a specific resource that provides benefits to firms [35]. According to the RBV theory, an excellent firm’s culture is usually a precious and unique one that is unable to be copied and replaced, and that can be deemed to be one of its core resources [36]. Green CSR strengthens the importance of proactive actions to handle environmental problems, and requires the development of innovative working methods, innovative products, services, processes, and so on [37,38]. Therefore, green CSR is considered to be a type of organizational ethical culture that distinguishes an organization from others in terms of pursuing competitive advantages and long-term development. From the RBV, it can be concluded that green CSR can stimulate firms’ proactive environmental actions, which may develop firms’ abilities to innovate and further facilitate firms’ innovation activities. As Russo and Fouts (1997) pointed out, RBV provides a solid foundation underpinning the argument that environmental performance positively relates to performance [39]. Following the foregoing theoretical logic, the hypothesis is formulated as the following:

H1: Green CSR is positively associated with innovation performance.

### 2.2. The Moderating Role of Public Visibility

Public visibility reflects the extent to which firms’ actions are observed by stakeholders. Public visibility plays as a pre-condition for stakeholders to respond to firms’ behaviors. The recent literature indicates that public visibility is generally correlated with stakeholders’ positive responses, including increased attractiveness of community stakeholders and favorable evaluations of regulatory stakeholders [40,41,42]. Based on this understanding, we propose that public visibility may moderate the relationship between green CSR and innovation performance.

The stakeholder theory argues that the success of firms relies on their capability to involve interest groups’ CSR desires in their business strategy [43]. While global stakeholders, particularly the community ones, are very concerned with green CSR nowadays, firms are expected to acknowledge green CSR and operate in green manners. In other words, awareness of firms’ green CSR not only increases community stakeholders’ identification, but also the intent of community stakeholders to commit personal resources to the benefit of the firm [44]. As the level of public visibility increases, firms that are responsible for their business conduct can receive broad attention of more community stakeholders [45]. Under this circumstance, firms can create more efficient channels for knowledge flow and obtain knowledge [46]. The more involuntary knowledge flows thus facilitate green CSR translate into innovation outcomes [47].

On the other hand, because visible firms can draw more attention, public visibility can also help regulatory stakeholders to judge whether firms’ green CSR meets their expectations [17]. Firms with greater public visibility may indicate conformity to normative pressures and show that they are considering the environmental impact on other interest groups instead of just thinking of personal benefits [1,48,49]. It is more likely that the greater the public visibility, the more regulatory stakeholders know legitimacy of firms, and the higher standard of firm citizenship. Thus, firms with high level of public visibility can better build, maintain, or enhance relationships with multiple regulatory stakeholders, whereby it is easier for them to access financial capital and preferential political supports [50]. By doing so, firms can engage in more innovation activities to create new products, new processes, and new ways of operating.

The contingency role of public visibility in firm performance has also been identified by previous research. For example, the research of Servaes and Tamayo (2013) shows that the relationship between CSR and firm value is stronger for firms with high customer awareness, as proxied by advertising expenditures [51]. Wang and Qian (2011) argue that public visibility affects stakeholder response to corporate philanthropy, which determines the variance of the relationship between corporate philanthropy and financial performance [52]. Based on the theoretical arguments and empirical results, it can be concluded that the more public visibility that firms have, the more benefits they will obtain from green CSR. Hence, the next hypothesis is formulated.

H2: Public visibility positively moderates the relationship between green CSR and innovation performance so that the positive effect of green CSR on innovation performance intensifies with high public visibility.

### 2.3. The Moderating Role of Firm Transparency

Firm transparency refers to the quality of a company’s disclosure of relevant important information regarding its business operation and management to the general public [53]. Increasing firm transparency is of great significance to the level of firms’ openness to their stakeholders, and it is an effective mechanism for decreasing information asymmetry between firms and stakeholders. Following this assumption, we propose that firm transparency may moderate the relationship between green CSR and innovation performance.

Firm transparency is achieved by information disclosure to meet the reasonable needs and interests of community stakeholders through constant communications. Firms showing greater transparency are assumed to devote substantial time and resources to providing sufficient information to the public in a timely manner [54], and this facilitates the communication of values and norms between firms and community stakeholders. Thus, firm transparency allows firms to build trustful relationship with community stakeholders. Under this circumstance, firms with higher transparency can convey the image of a reputable organization so that community stakeholders will have strong willingness to support [55]. When the firm transparency is high, the effective social knowledge exchange rises, and these strong ties are more likely to expend effort to ensure that firms can sufficiently understand and put into use newly acquired knowledge, so green CSR can initiative innovation more effectively.

On the other side, firms seeking to gain or maintain political legitimacy also have an incentive to use transparency strategies. The level of availability and accessibility of information to its regulatory stakeholders is a fundamental requirement for the operating of firms, and the greater supply of firm transparency can signal firms’ long-term promises to environmental protection. Those firms that have a high degree of firm transparency in their annual reports are believed to have adopted more proactive environmental strategies, which can meet the legal requirements set by regulatory stakeholders [56]. Firm transparency leads regulatory stakeholders to interpret and respond positively in the information asymmetry context, which further helps firms construct high quality interrelationship with regulatory stakeholders. Therefore, firms with high transparency can prevent themselves from government interference and enjoy institutional support. The tangible and intangible resources acquired by the firms enable green CSR to produce innovation process and activities more efficiently and effectively. For instance, Du et al. (2011) confirm the critical role of consumer trust in the linkage between CSR and competitive advantage [57]. Amores-Salvadó et al. (2014) reveal that green corporate image, the impression of a firm in the mind of their stakeholders, moderates the relationship between environmental product innovation and firm performance [17]. Extending the previous research, it can be postulated that the more firm transparency that firms have, the more benefits they will obtain from green CSR. Hence, the next hypothesis is formulated.

H3: Firm transparency positively moderates the relationship between green CSR and innovation performance so that the positive effect of green CSR on innovation performance intensifies under high levels of firm transparency.

## 3. Methods

### 3.1. Data and Sample Selection

Our samples are selected from the Chinese listed firms between 2006 and 2015. The samples are initially taken from the Shenzhen stock exchange website, and then we searched basic information regarding each firm. This paper subjectively measures the quantity of the obtainable green CSR data. The green CSR was obtained from the firms’ annual reports, environmental reports, and the firms’ websites. The advertising, sales growth, firm size, leverage, and return on assets data, etc., come from firms’ annual reports and China Stock Market and Accounting Research (CSMAR) data base. The firm transparency data were obtained from the Shenzhen stock exchange website. We also combined State Intellectual Property Office of the People’s Republic of China and China National Knowledge Infrastructure to obtain innovation performance data. The paper excludes Special Treatment (ST) firms due to the fact that they are in a financially abnormal status, and thus they are not suitable to analyze. Moreover, this paper also deleted a few observations as a result of their lack of complete data for analysis. Overall, it selected 390 valid observations.

### 3.2. Measurement

Following previous research, this paper uses green CSR disclosure to measure green CSR. Firm self-disclosure scores on green CSR are usually derived from the content analysis of firm documents such as annual reports, corporate environmental reports, and corporate websites [22,58,59]. In our data collection effort, we depended on the data from firms’ annual reports, CSR reports, green CSR reports (when available), and corporate websites. Our final green CSR measure comprises 17 items, which are environmental management system (qualitative and quantitative), energy saving (qualitative and quantitative), emission reduction (qualitative and quantitative), garbage disposal (qualitative and quantitative), beneficial products and services, pollution prevention, renewable energy, resources recycling, water efficiency, sustainable packaging, process improvement, green innovation, and environmental investment.

Public visibility is measured by firm advertising intensity [52]. Obviously, wide advertisement can absorb more attention of outside interest groups [32]. A company with intensive advertisement is more popular to its interest groups, who can have more recognition of its green CSR. Thus, the intensity of a firm’s advertising can be a proxy index of its public visibility. A firm’s advertisement intensity is calculated as the ratio of advertising costs to selling, which reflects the willingness of firms to invest more in advertising and selling so as to do better than others.

To capture a firm’s level of the firm transparency of firm-specific information concerning publicly listed firms to the stakeholders, this paper uses the evaluation score on information disclosure of Shenzhen stock exchange to be the proxy index of firm transparency [54]. The scores are used to assess a firm’s communication effectiveness with its stakeholders and its degree of information disclosures needed for stakeholders to make decisions.

Patent data is extensively used to measure innovation performance [60]. Thus, the number of successful patent applications filed during each year is used as the dependent variable in this paper. In addition to the green CSR related variables, several control variables are added to explain firm innovation performance, including sales growth, firm size, leverage, and return on assets (ROA). Sales growth is used as a control variable because previous studies report that high growth firms are inclined to increase their share of exploration [61]. Firm size, measured by log assets, has been regarded as an important variable in researches on the innovation performance [62]. Leverage is often used as a proxy variable for firms’ risk level. If a firm’s leverage is high, it may be threatened with innovation distress [63]. We also include ROA in the model because it may influence a firm’s motivation to innovate [64,65].

## 4. Results and Discussion

### 4.1. Descriptive Statistics

Table 2 reports the descriptive statistics and correlations of the key variables. It can be noted that, overall, no correlations among the variables are high enough to raise major concerns about multicollinearity. As expected, green CSR is significantly and positively correlated with innovation performance. Furthermore, public visibility and firm transparency are significantly and positively correlated with innovation performance, which may provide an insight into our basic theoretical position: public visibility and firm transparency may play moderating roles in the relationship between green CSR and innovation performance.

### 4.2. Regression Analysis Results

To test moderating effects of public visibility and firm transparency, we followed the usual procedure of testing moderating effects by first including the independent and control variables, then adding interaction variables, and at last running full model with all variables. We measure variance inflation factors (VIF) of all regression models to check multicollinearity. The range of VIF is below the typical cut-off of 10. Table 3 shows the results.

From Table 3 we can know that the level green CSR is a strong predictor of innovation performance. The coefficient is consistently positive and significant in Model 2 (β = 0.245, *p* < 0.01) and Model 8 (β = 0.475, *p* < 0.01). Therefore, Hypothesis 1 is valid. This result agrees with the research of Flammer (2013), indicating that green CSR generates new and competitive resources for firms [1]. Firms voluntarily integrating environmental concerns in their business operations may capture more valuable resources, which can be applied to innovation activities.

In Model 5 and Model 8, the coefficient of the interactions involving green CSR and public visibility is both positive and significant (β = 0.328, *p* < 0.01; β = 0.330, *p* < 0.01). Therefore, Hypothesis 2 is supported. Similarity, in Model 7 and Model 8, the coefficient of the interactions involving green CSR and firm transparency is also both positive and significant (β = 0.252, *p* < 0.01; β = 0.245, *p* < 0.01). Therefore, Hypothesis 3 is supported. Previous research has revealed that the quality of social interactions establishes a key context for CSR research [44]. For instance, Fry et al. (1982) point out that public contact can strengthen the relationship between CSR engagement and outcomes [66]. Our study shifts the emphasis and completes such assertions by showing that public visibility and firm transparency can also provide important contingencies for green CSR. Through reciprocal connections with stakeholders built by high quality of public visibility and firm transparency, firms are more likely to be known, and as a result they can receive more inputs from various stakeholders. It then follows that firms with high quality of public visibility and firm transparency are likely to capture more value from green CSR.

**Figure 1:**
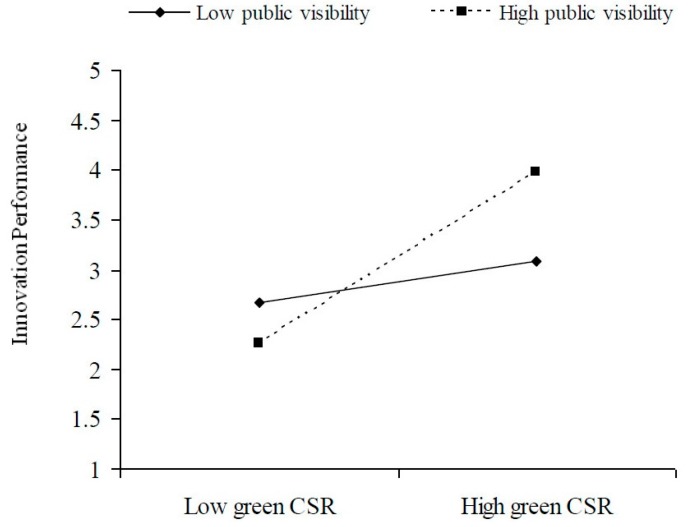
The moderating effect of public visibility.

**Figure 2:**
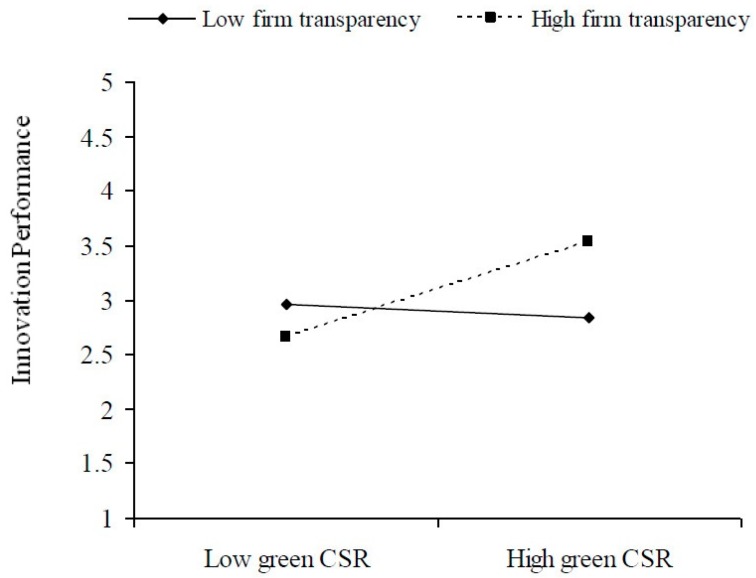
The moderating effect of firm transparency.

To explain these findings better, this paper utilized Aiken and West’s [67] approach to find the significant interaction effect of public visibility and firm transparency (Figure 1 and Figure 2). The figures show that at high levels of public visibility and firm transparency, the relationship between green CSR and innovation performance is more positive. In contrast, at low levels of these variables the relationship between green CSR and innovation performance tends to become less positive or negative.

## 5. Conclusions

This paper aims to test the correlation between green CSR and innovation performance considering the moderating roles of public visibility and firm transparency. Our quantitative analysis finds that green CSR has a positive relation with innovation performance that is parallel to the Porter Hypothesis [68]. To take responsibility to implement environmentally friendly activities, companies need to become more innovative than those choosing not to [69,70]. Green CSR can trigger innovation through improving products, producing best-practice technologies, and developing new ways of using best-practice technologies [71]. The sustainable approach towards environment fosters innovation activities, which means that greater concern for green CSR can always lead to the increasing of innovation performance.

Intriguingly, the results also suggest that the positive relationship between green CSR and innovation performance becomes stronger with higher public visibility and firm transparency, which is consistent with the stakeholder theory that the more green CSR is perceived by stakeholders, the more potential benefits are achieved by obtaining necessary stakeholder resources and supports [1]. The availability of information is a key determinant for stakeholders to better understand firms’ environmental behavior. Stakeholders would like to assess the company positively when they have access to a large amount and high quality of information to judge green CSR practices [72]. The public visibility, which means voluntarily disclosing more environmental information, can inform stakeholder firms’ proactive environmental strategy, and provide an open door for stakeholders to observe their green CSR. Similarly, firm transparency can decrease the negative impact of information asymmetry and help stakeholders to interpret and evaluate green CSR more easily and accurately [73]. When the level of public visibility and firm transparency is high, firms can maintain an extensive and closer relationship with stakeholders, and achieve more firms’ legitimacy, and thus strengthen the positive effects of green CSR on innovation performance.

This paper improves upon the existing literature on green CSR in three aspects. First, for green CSR literature, this paper enhances our perception of the correlation between green CSR and innovation performance. It found out the two mechanisms—deducing positive stakeholder responses and obtaining political legitimacy—that lead to the link between green CSR and innovation performance. These findings help establish a more solid theoretical foundation for the correlation between green CSR and innovation performance. Our findings indicate that green CSR must be further investigated to reveal the related issues.

Second, we also have had a further explanation for the conditions in which the correlations between green CSR and innovation performance are intensified or weakened through stakeholder perspective. By connecting theory on CSR and stakeholders, our study suggests that innovation performance depends on the joint effect of CSR and social exchange. That is, public visibility and firm transparency have significant moderating effects on the relationship between green CSR and innovation performance, which implies that a firm’s own features have a significant influence in deciding the degree of benefit from green CSR. The results of the moderating impact of public visibility and firm transparency provide a better knowledge of the underlying mechanisms through which green CSR is correlated to innovation performance. Thus, this study goes beyond recent conversations concerning green CSR and further opens an agenda for other moderating conditions.

This paper also contributes to the resource-based view of the literature. It argues that the importance of green CSR as valuable, rare, imperfectly imitable, and non-substitutable resource that can lead to a competitive advantage. Thus, this paper integrates the resource-based view framework with CSR and provides a new perspective for determining competitiveness. Furthermore, there have been calls for the further comprehension of the boundary conditions of the micro-foundations of the resource-based view [35], and it is a response providing insights into how firms access the resources by identifying the influence of public visibility and firm transparency on the value realized by green CSR.

This paper has some practical implications as well. First, there have been debates about whether firms should engage in green CSR. This paper suggests that firms should invest more resources to enhance green CSR, because it is positively associated with innovation performance. Hence, firms should incorporate environmental thinking into corporate philosophy and commitments, and ensure the concept of sustainable development is deeply rooted in the corporate culture. Second, this paper further demonstrates that firms do not all equally benefit from green CSR. Public visibility and firm transparency can generate meaningful signals to stakeholders, and strengthen the positive relationship between green CSR and innovation performance. Thus, firms that are already active in green CSR should improve their information sharing awareness, manage the interface between firm and stakeholders, and respond accordingly in shaping organizational policies towards stakeholder requirements to ensure their green CSR is perceived by stakeholders.

Although our study provides important insights regarding the role of green CSR as an antecedent of innovation performance, it can be extended in several ways. First, a limitation of this study is our measurements of some variables. For example, although content analysis had been used to measure green CSR extensively, it cannot capture a firm’s actual achievement related to green activities. Further research might set out to construct more robust measures to reflect a firm’s green CSR, such as developing a scale. Also, even though the patent dataset is widely used to measure innovation performance, not all firms hold patents to protect their knowledge or technology [74,75,76]. Therefore, the results would be greatly strengthened if future studies could directly measure innovation performance. Second, another limitation of this study is that we do not address the specific mediating mechanisms of the link between green CSR and innovation performance. Therefore, in order to fully benefit from green CSR in terms of enhancing innovation performance, future research is needed to investigate the mechanism for that. Third, we analyze the contingency contexts of the relationship between green CSR and innovation performance only in terms of public visibility and firm transparency. Future studies could contribute by advancing our attempt to analyze other types of moderators, which would provide a more complete understanding of how green CSR affects innovation performance. Last but not least, the sample was limited to Chinese publicly listed firms. It would, though, be interesting to examine these research questions in a multi-country context that provides more variation in the level of legal and financial sophistication [77,78].

## Figures and Tables

**Table 1:** Summary of studies on the relationship between green CSR and firm performance.

Study	Operational Definition of Green CSR	Performance			Operational Definition of Performance
		Environmental	Marketing	Financial	
Chuang and Huang [12]	Multidimensional construct: disclosed governance, credibility, and environmental performance.	√			The outcomes of business’s environmental protection and environmental management policy.
Johns and Khojastehpour [13]	Climate responsibility and natural resource utilization.		√		Stakeholders’ perceptions, attitudes, and esteem.
Rashid et al. [14]	Multidimensional construct: environmental philanthropy, community involvement, and customer wellbeing.		√		Positive consumer behavior.
Sáez-Martínez et al. [15]	Likert-type variable reflects pro-environmental attitudes.		√		Sales growth.
Iwata and Okada [16]	Reducing waste and greenhouse gas emissions.			√	ROA, ROI, ROIC, Tobin’s Q.
Amores-Salvadó et al. [17]	Likert-type variable demonstrates firm’s environmental concern.			√	ROA, ROS, and ROCE growth relative to competitors.
Wei et al. [18]	Likert-type variable pertains firms’ green behavior.			√	ROA, ROI, profit margins, sale, market share, and profit growth.

Notes: ROA represents for return on assets; ROI represents for return on investment; ROIC represents for return on invested capital.

**Table 2:** Descriptives Statistics and correlations.

Variable	Mean	SD	1	2	3	4	5	6	7	8
1 Sales growth	0.228	0.844	1							
2 Firm size	10.031	0.456	−0.015	1						
3 Levarage	0.549	0.187	0.009	0.279 ***	1					
4 ROA	0.052	0.068	0.121 **	−0.026	−0.609 ***	1				
5 Green CSR	7.149	8.842	−0.050	0.359 ***	0.011	−0.041	1			
6 Public visibility	0.009	0.017	−0.024	0.016	−0.244 ***	0.284 ***	−0.104 **	1		
7 Firm transparnecy	3.138	0.626	−0.008	0.183 ***	−0.200 ***	0.217 ***	0.106 **	0.110 **	1	
8 Innovation performance	84.151	456.035	−0.028	0.290 ***	0.121 **	−0.052	0.311 ***	0.027 **	0.137 ***	1

Notes: ** *p* < 0.05, *** *p* < 0.01; ROA represents for return on assets.

**Table 3:** Regression Analysis Results.

Variable	Model 1	Model 2	Model 3	Model 5	Model 6	Model 7	Model 8
Sales growth	−0.021	−0.015	−0.014	−0.014	−0.013	0.003	0.004
Firm size	0.281 ***	0.181 ***	0.180 ***	0.175	0.158	0.161	0.152
Levarage	0.027	0.074	0.077	0.075	0.094	0.062	0.068
ROA	−0.026	0.009	0.007	0.002	−0.001	0.021	0.012
Green CSR		0.245 ***	0.246 ***	0.532 ***	0.242 ***	0.186 ***	0.475 ***
Public visibility			0.012 **	0.119 **			0.117 **
Firm transparnecy					0.102 **	0.098 **	0.115 **
Green CSR × Public visibility				0.328 ***			0.330 ***
Green CSR × Firm transparnecy						0.252 ***	0.245 ***
R^2^	0.087	0.137	0.137	0.160	0.147	0.204	0.227
F	9.121 ***	12.227 ***	10.174 ***	10.374 ***	10.963 ***	14.025 ***	12.380 ***

Notes: ** *p* < 0.05, *** *p* < 0.01; ROA represents for return on assets.
